# Wireless Resonators With Coupled Versus Decoupled Units: Which Enhances Local SNR of RF Receive Arrays Better?

**DOI:** 10.1002/mrm.70170

**Published:** 2025-11-05

**Authors:** Ming Lu, Haoqin Zhu, Ruilin Wang, Xinqiang Yan

**Affiliations:** ^1^ Vanderbilt University Institute of Imaging Science, Vanderbilt University Medical Center Nashville Tennessee USA; ^2^ College of Nuclear Equipment and Nuclear Engineering Yantai University Yantai Shandong China; ^3^ Sino Canada Health Institute Inc. Winnipeg Manitoba Canada; ^4^ Department of Radiology and Radiological Sciences Vanderbilt University Medical Center Nashville Tennessee USA; ^5^ Department of Electrical and Computer Engineering Vanderbilt University Nashville Tennessee USA

**Keywords:** g‐factor, MRI, passive resonator, signal‐to‐noise ratio (SNR), wireless resonator

## Abstract

**Purpose:**

To compare two identically sized wireless resonator designs, one with strongly coupled units and the other with decoupled units, for their ability to enhance receive performance in MRI when used with local receive arrays.

**Methods:**

Both wireless resonator designs were fabricated and experimentally evaluated for detuning efficiency, SNR improvements, and parallel imaging performance (g‐factor) at 1.5 T. They were used alongside a 12‐channel head receive array, with the standard body coil serving as the RF transmitter.

**Results:**

Experimental data showed that the wireless resonator with decoupled units consistently outperformed that with coupled units, with up to threefold improvement in SNR and a reduction of maximum/average g‐factor from 4.6/1.8 to 3.1/1.3. Notably, compared to the original receive array (maximum/average: 3.9/1.7), the decoupled design further improved the g‐factor, highlighting superior performance in accelerated imaging.

**Conclusion:**

Wireless resonators with decoupled units offer significant advantages in improving MRI image quality and parallel imaging performance over their coupled counterparts. Their ease of detuning and pronounced gains in SNR and g‐factor make them a compelling choice for wireless resonator designs.

## 1 Introduction

The signal‐to‐noise ratio (SNR) is one of the most critical parameters in MRI, as it directly determines spatial resolution and imaging quality. From a hardware perspective, optimizing the radiofrequency (RF) receive coils is a well‐established method to enhance SNR. In modern MRI systems, multi‐element receive arrays have become the standard due to their superior SNR and enhanced encoding capabilities for accelerated imaging [1, 2, 3, 4, 5, 6, 7, 8].

Another effective strategy to enhance imaging performance, particularly local SNR, involves placing passive inserts within the receive arrays. These inserts can be broadly grouped into two categories: dielectric pads [9, 10, 11, 12] and inductively‐coupled resonators [13, 14, 15, 16, 17, 18, 19, 20, 21, 22, 23, 24, 25, 26, 27, 28, 29, 30, 31, 32]. Inductively coupled resonators, in contrast, consist of RF resonator units that interact with the primary coil via inductive or magnetic resonance coupling. Often termed wireless resonators, wireless coils, inductively coupled coils, or passive resonators/antennas, this paper adopts the term wireless resonators for consistency.

In addition to wireless resonators, such as loop resonators and ladder resonators that have been widely used in MRI, dipole antennas [19, 33] and metamaterials [34, 35, 36, 37, 38, 39, 40], originally developed in the microwave domain, have recently been applied to MRI. The underlying mechanisms of wireless dipoles [19, 33] and metamaterials are believed to be similar to those of wireless resonators, which have been utilized in MRI for over 40 years [13, 14]. However, dipoles are non‐resonant structures and are primarily employed as reflectors [19, 33]. Metamaterials generally utilize distributed elements rather than lumped capacitors, and their layouts and structural configurations differ significantly from those ladder or loop resonators [40].

Historically, wireless resonators have predominantly been coupled with the body coil, showing improved performance compared to the body coil alone [13, 19, 22, 24, 25, 28]. However, since the body coil is primarily used for RF transmission, it inherently offers low SNR for reception. The primary workhorse for RF reception remains the local receive array. Recently, detunable wireless resonators have demonstrated significant advantages when integrated with local receive coil arrays, particularly in enhancing local SNR [26, 27, 30].

Wireless resonators can be further classified based on inter‐unit isolation into two categories. The first category includes resonators with coupled units. Owing to the strong coupling among units, such resonators act as a unified structure but exhibit multiple resonant modes, with the 𝑚 = 1 mode typically being the desired one [28, 41]. A well‐known example is the birdcage resonator or ladder resonator. The second category consists of resonators with highly decoupled units, where each unit is isolated, resulting in a single resonant mode [26]. Although decoupled and coupled wireless resonators differ in their inter‐element coupling mechanisms, both interact inductively with the main receive array and have demonstrated improved local SNR as passive inserts [26, 41]. However, a comprehensive evaluation of their relative performance is lacking, and it remains unclear which type of wireless resonator performs better under practical conditions. For the first time, this work investigates these two types of wireless resonators of identical size through comprehensive MRI experiments.

## 2 Methods

**FIGURE 1:**
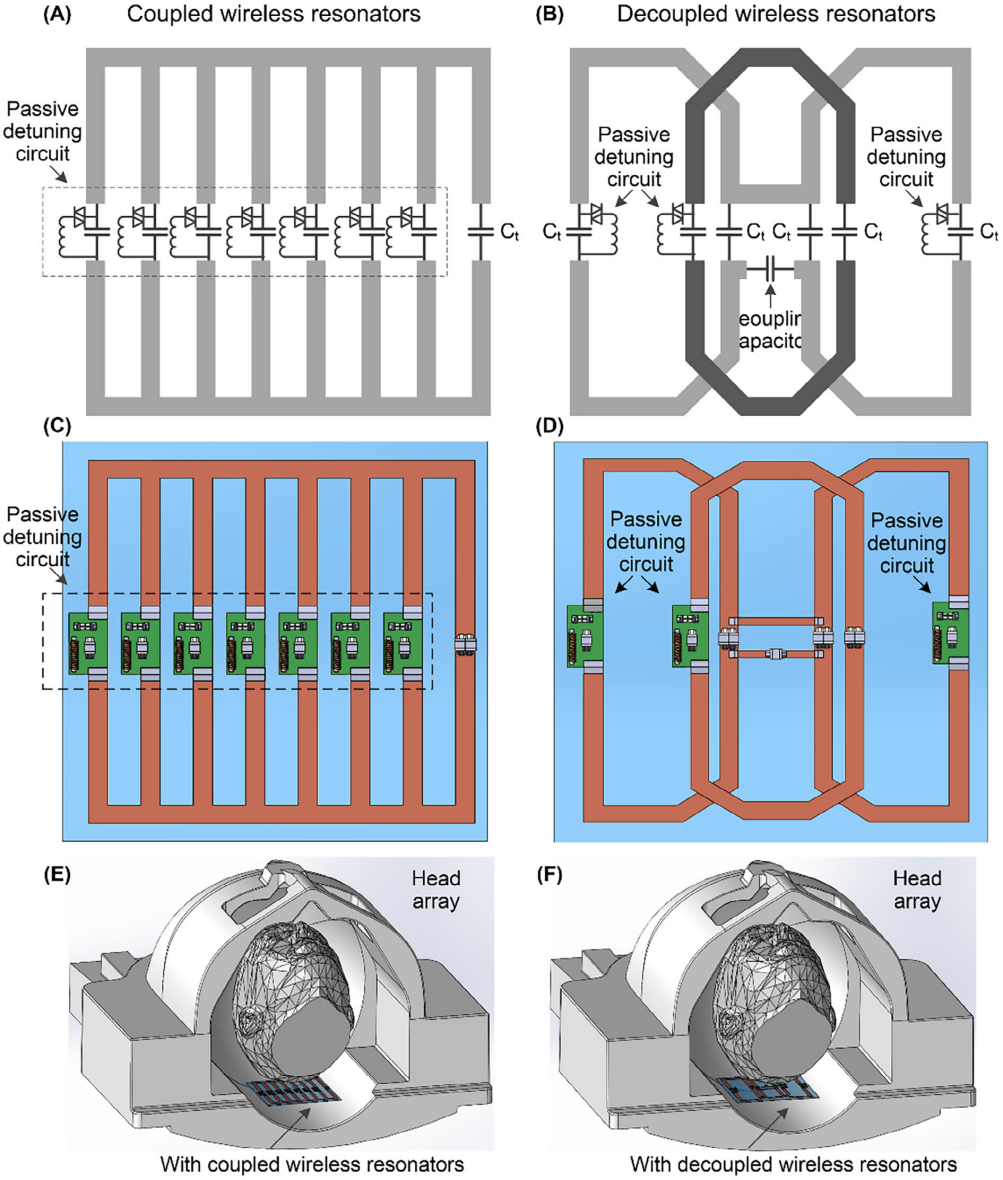
(A, B) Circuit diagrams of the constructed wireless resonators with coupled and decoupled units. (C, D) CAD and PCB design of these wireless resonators. (E, F) Illustration of the relative position of the head array and wireless resonators.

In this study, the wireless resonator with strongly coupled units is designed as a non‐closed ladder resonator, also known as a partial birdcage resonator (Figure 1A,C) [41]. This low‐pass, 7‐unit design incorporates capacitors of identical value positioned along the rungs. In contrast, the wireless resonator with well‐decoupled units is configured as a single‐row array of three well‐decoupled loops (Figure 1B,D). For the 3‐loop wireless array, adjacent loops are overlapped, while non‐adjacent loops are decoupled using a bridging capacitor network. Both wireless resonators share identical overall dimensions of 10 cm in length and 10 cm in width. To ensure consistency in the number of units between the coupled and decoupled designs, we also fabricated a three‐unit coupled ladder resonator and showed the results in Supporting Information. However, the majority of results for coupled‐unit resonators focus on the seven‐rung design, as it represents a more optimal configuration compared to the three‐unit design [41].

The wireless resonators were fabricated to work with a 12‐channel head array at 1.5 T (Siemens Sempra, Siemens Medical Solutions), as depicted in Figure 1E,F. Each rung in the wireless ladder resonator and each loop in the wireless array incorporates a passive detuning circuit (add‐on inductor and crossed diodes) for detuning.

During the RF transmission phase, the built‐in body coil was utilized, with both the 12‐channel head array and the wireless resonators detuned. During the RF reception phase, the 12‐channel head array was used in conjunction with the wireless resonators. The 𝑚 = 1 mode of the wireless ladder resonator and the single resonant mode of the wireless array were tuned to 63.67 MHz, corresponding to the scanner's Larmor frequency.

We evaluated the wireless resonator's detuning performance by comparing the B_1_^+^ map (with the same input power) on a cylindrical phantom (diameter of ∼15 cm, 7300‐mL water with 9.1 g NiSO_4_ × H_2_O and 19.1 g NaCl) with that acquired without wireless resonators. The B_1_^+^ mapping was acquired using the scanner's default TurboFLASH method [42], with the following parameters: TR/TE = 300/14 ms, field of view (FOV) = 250 × 250 mm^2^, matrix = 128 × 128, and slice thickness = 10 mm. Additionally, we obtained RF power amplitude to achieve a 90‐degree flip angle.

We assessed the SNR and g‐factor using the same phantom. Experiments were conducted with the head array alone, and in combination with wireless resonators of either coupled or decoupled units. The pulse sequence used for SNR evaluation was Gradient Recalled Echo (GRE) with the following parameters: axial orientation, FOV = 200 × 200 mm^2^, TR/TE = 300/10 ms, flip angle (FA) = 60°, matrix = 256 × 256, bandwidth (BW) = 130 Hz/pixel, slice thickness = 3 mm, and number of averages = 1.

In addition to phantom images, T1‐weighted (T1W) images of a healthy volunteer were also acquired using different setups. The imaging protocols were as follows: sagittal slice, TR/TE = 5520/88 ms, FA = 150°, FOV = 223 × 230 mm^2^, matrix = 298 × 384, BW = 191 Hz/pixel, slice thickness/slice gap = 2/2.3 mm, 27 slices, number of averages = 3. Safety tests for gradient‐induced and RF‐induced heating were conducted before human imaging. All experimental procedures were approved by the local institutional review board (Approval ID: YKD202402001), and participants provided informed written consent. During the MRI experiments, the wireless resonators were wrapped around the bottom of phantom or human head.

## 3 Results

**FIGURE 2:**
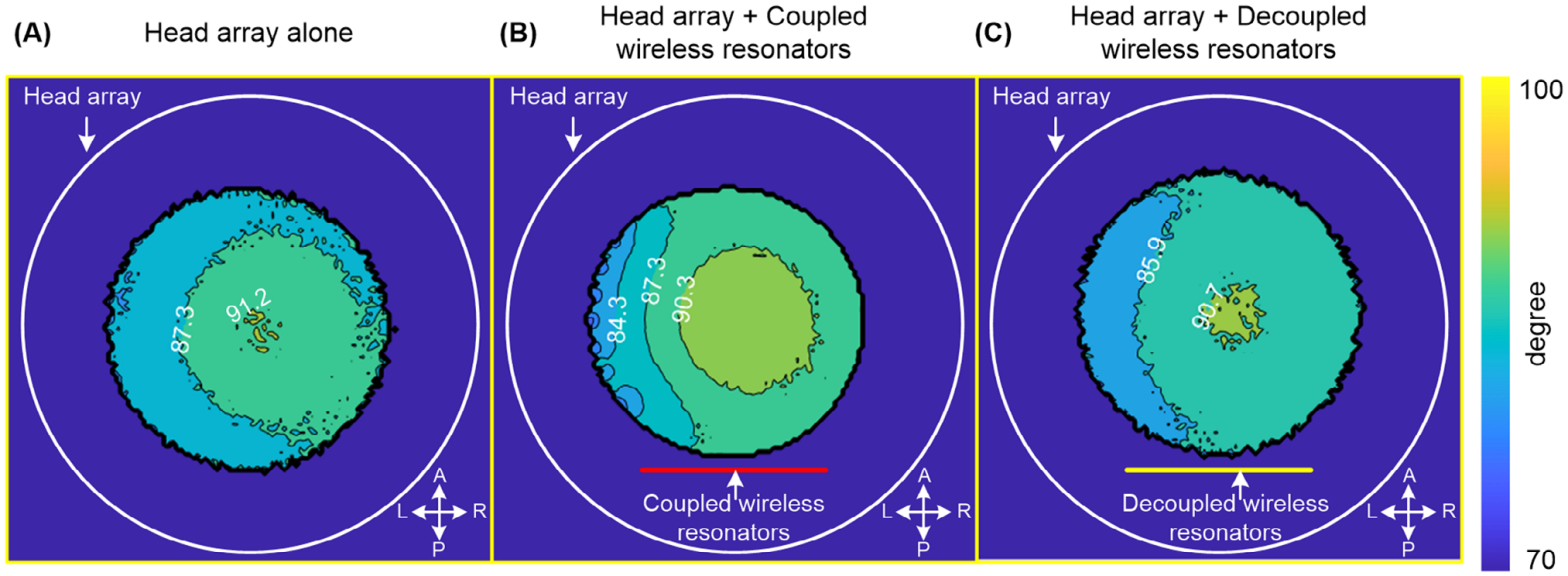
Measured axial B_1_^+^ maps for the 12‐channel head array alone (A), with wireless ladder resonator (B), and with wireless 3‐loop resonator (C). Axial B_1_^+^ maps were measured on a cylindrical phantom with the same input power.

Figure 2 shows axial B_1_^+^ maps on the phantom acquired under identical input power with different configurations. No B_1_^+^ distortion was observed with either the wireless ladder or the 3‐loop resonator. Compared to the B_1_^+^ maps without wireless resonators, B_1_^+^ differences remained under 2%. Further B_1_^+^ calibration confirmed that the RF power required for a 90° flip angle showed minimal variation: 284.1 V (no resonator), 281.9 V (ladder), and 284.0 V (3‐loop).

**FIGURE 3:**
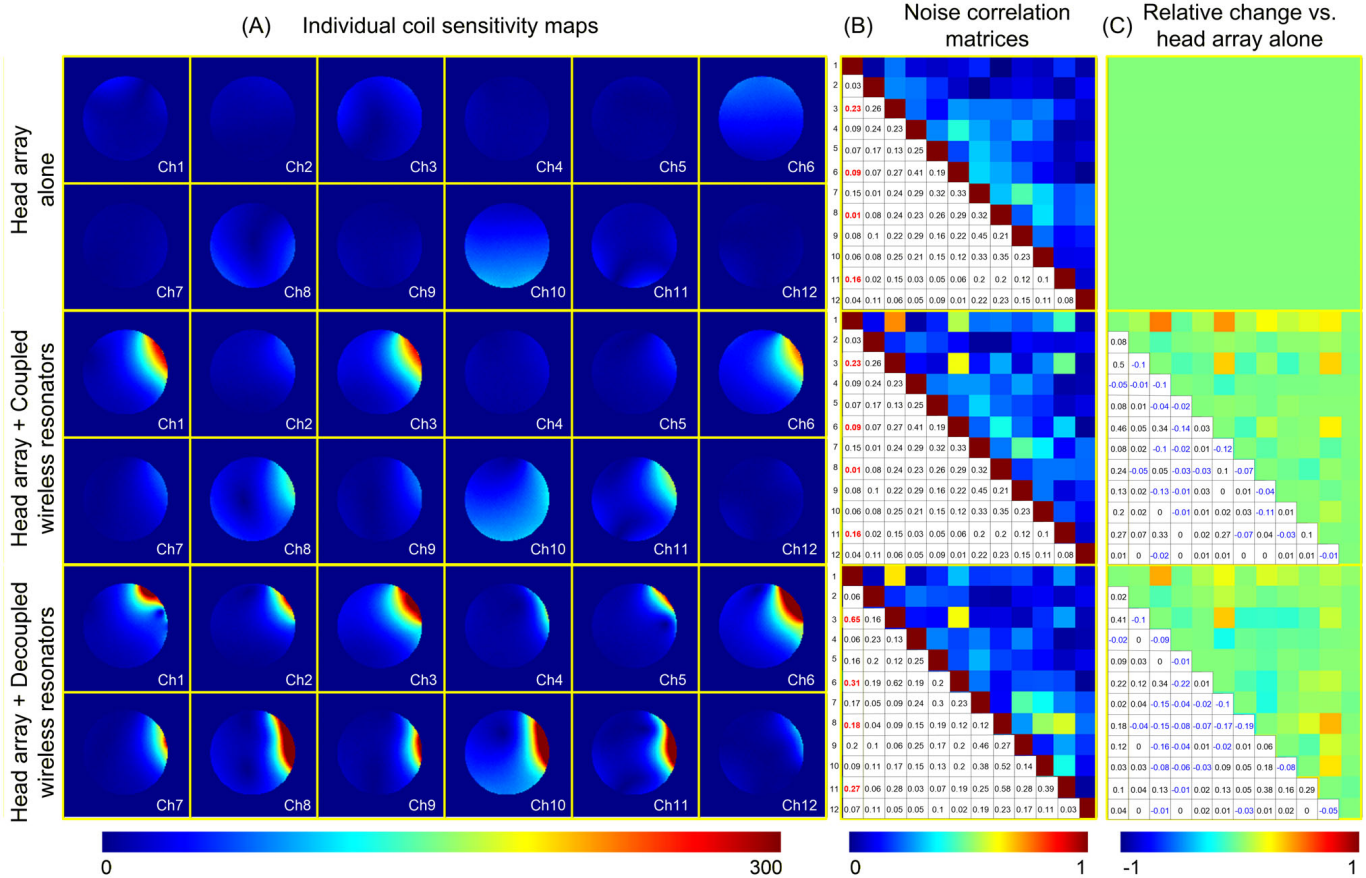
(A) Individual coil sensitivity maps of the 12‐channel head array: Alone (top row), with the wireless ladder resonator (middle row), and with the wireless 3‐loop resonator (bottom row). (B) Noise correlation matrices for the 12‐channel head array alone, with wireless ladder resonator, and with wireless 3‐loop resonator. (C) Changes relative to the standalone 12‐channel array.

Figure 3A compares the individual coil sensitivity maps of the 12‐channel head array under three configurations: standalone, with ladder resonator, and with 3‐loop array. Sensitivity maps were calculated by dividing signal intensity by the standard deviation of the corresponding noise for each coil. With the coupled ladder resonator, coils 1, 3, 6, 8, and 11—located closest to the wireless units—showed significant sensitivity enhancement due to strong mutual inductive coupling. This coupling caused their sensitivity profiles to align with the resonator, reducing pattern diversity, which can lead to increased noise correlation and a degraded g‐factor. In contrast, the decoupled 3‐loop resonator improved sensitivity not only in nearby coils (1, 3, 6, 8, 11), but also across the array. Due to the high isolation between its units, the coils primarily couple to individual resonators rather than the entire array, preserving unique sensitivity profiles. Near the wireless loops, this diversity even exceeds that of the original array.

Figure 3B shows the noise correlation matrices for the three setups, and Figure 3C highlights the changes relative to the standalone array. With the ladder resonator, strong inter‐unit coupling created a shared coupling pathway across multiple coils, resulting in overlapping sensitivity regions and a substantial increase in noise correlation. For example, the correlation between elements 1&3, 1&6, 1&8, and 1&11 rose from 0.23, 0.09, 0.24, and 0.16 to 0.74, 0.55, 0.24, and 0.44, respectively. The 3‐loop array also led to increased noise correlation, but to a much lesser extent (0.65, 0.31, 0.18, and 0.27 for the same pairs). As noted above, this is consistent with the preserved sensitivity diversity, owing to minimal shared coupling paths among the decoupled units.

**FIGURE 4:**
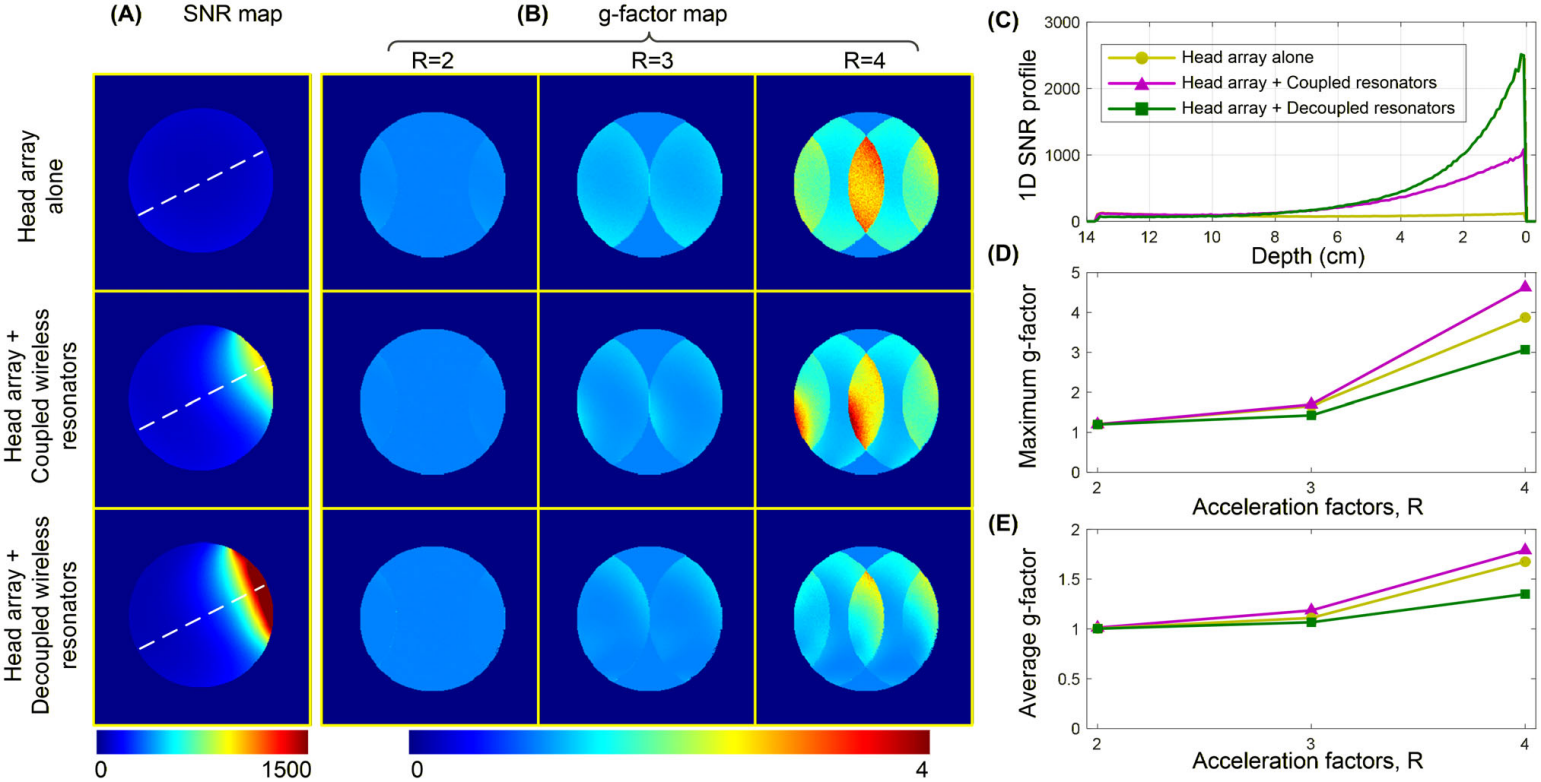
(A) Axial SNR maps of the 12‐channel head array alone, with the wireless ladder resonator, and with the wireless 3‐loop resonator. (B) SENSE g‐factor maps on the phantom with left–right acceleration factors (R) of 2, 3, and 4. (C) 1D SNR profiles along the indicated direction (white dotted lines in A). (D, E) Plots of maximum and average g‐factors for various setups.

Figure 4A compares the axial SNR maps using optimal coil combination [1, 43]. Figure 4C plots 1D SNR profiles along white dotted lines in Figure 4A. The comparison of two wireless resonator designs, the coupled‐unit wireless ladder, and the decoupled‐unit wireless 3‐loop array, revealed significant SNR improvements over the 12‐channel head array alone. At a depth of 2 cm, the wireless 3‐loop array achieved an SNR improvement of 10.6 times, compared to 6.8 times for the wireless ladder. At the center of the phantom, both resonators provided similar SNR enhancements relative to the head array, with improvements of 2.2 times. Notably, the wireless 3‐loop array exhibited superior performance over the wireless ladder at depths less than 6 cm, emphasizing the benefits of the decoupled design in optimizing local SNR. Figure S1 shows the SNR comparison for a 3‐unit wireless ladder resonator, which has the same number of units as the wireless decoupled array. However, its local SNR enhancement is only 3.5 times, significantly lower than that of the wireless 3‐loop array (10.6 times) or the wireless 7‐unit ladder resonator (6.8 times).

Figure 4B compares the g‐factor maps for left–right acceleration with R of 2, 3, and 4. A lower g‐factor signifies better parallel imaging performance, reducing noise during image reconstruction and ensuring a more optimal coil design for parallel imaging. Figure 4D,E illustrate the maximum and average g‐factors within the slice. The introduction of the wireless resonator with strongly coupled units (i.e., the wireless ladder) degraded the g‐factor performance of the 12‐channel head array. For instance, at *R* = 3 in the right–left direction, the maximum/average g‐factor values increased from 3.9/1.7 to 4.6/1.8. In contrast, the setup with the added wireless resonator with decoupled units (i.e., wireless 3‐loop array) demonstrated improved g‐factor performance compared to the original 12‐channel array. For example, at *R* = 3 in the right–left direction, the maximum/average g‐factor values were 3.1/1.3 for the wireless 3‐loop setup, compared to 3.9/1.7 for the original 12‐channel array. These results indicate that strongly coupled wireless units negatively impact the performance of accelerated imaging of the receive array, while decoupled wireless units can enhance this performance.

**FIGURE 5:**
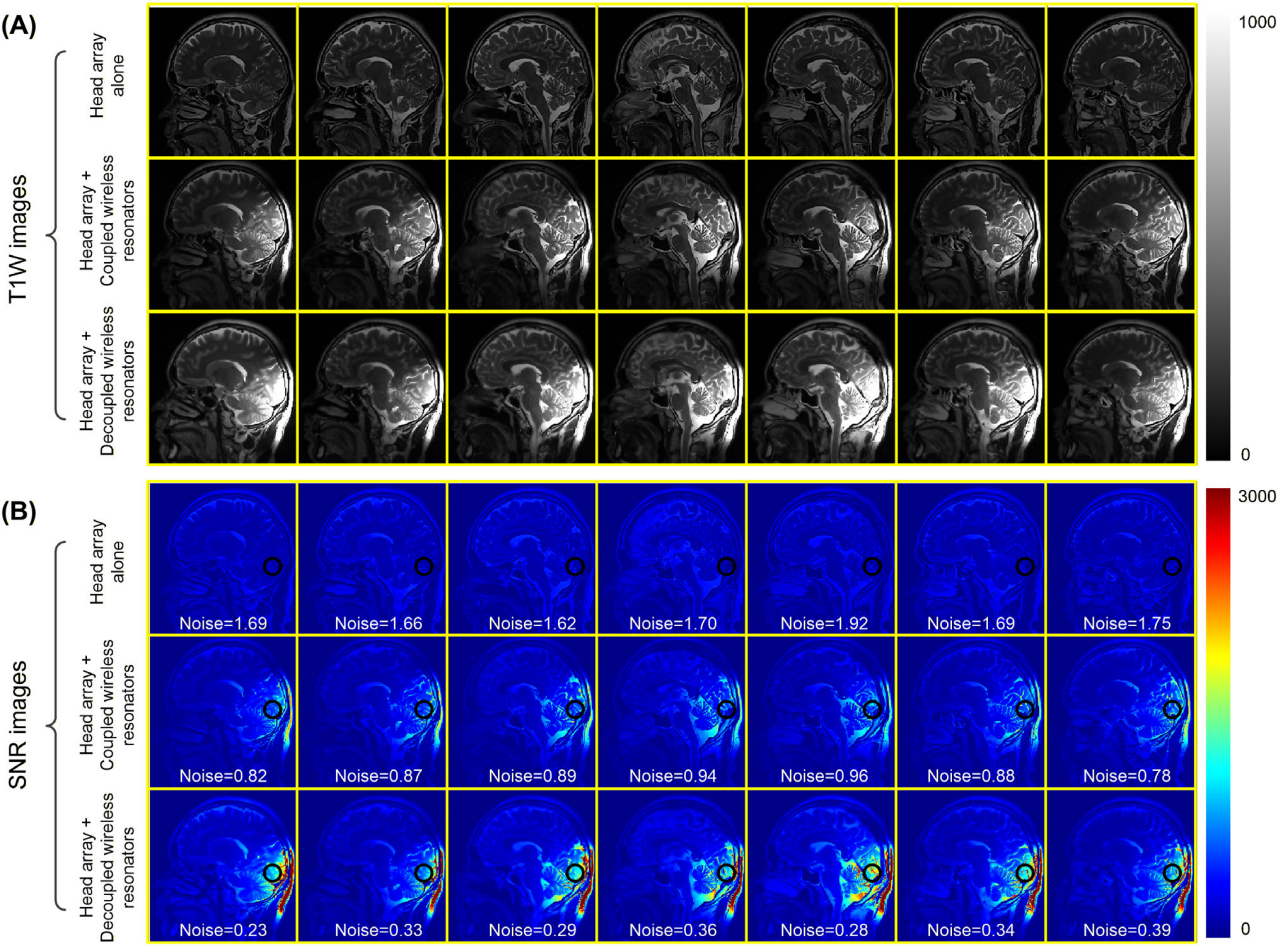
(A) Acquired T1W images in sagittal slices on a healthy volunteer using various setups. (B) Corresponding SNR maps derived from the T1W images.

Figure 5A presents the acquired T1W images in sagittal slices, while Figure 5B shows the corresponding SNR maps derived from these images. Consistent with the observations from the phantom MRI, the wireless ladder resonator exhibits greater SNR enhancement at the bottom of the human brain compared to the wireless 3‐loop configuration. Quantitatively, the local SNR improvement with the wireless ladder resonator is approximately 4.2‐fold, while the wireless 3 loops show an improvement of 12.8‐fold. The SNR calculation was based on a circular area shown in Figure 5B. Since these wireless resonators are 10 cm wide and do not cover the entire brain, the SNR enhancement is primarily localized to nearby areas, such as the occipital lobe and cerebellum. Unlike the local SNR, the introduction of wireless resonators (either the ladder or 3‐loop design) exhibits similar or slightly higher central SNR compared to the 12‐channel array alone.

While this localized improvement in imaging quality leads to some degree of reception inhomogeneity, it is important to note that these images were reconstructed without the application of filters, windowing, or normalization algorithms. Modern imaging workflows have significantly improved the management of reception inhomogeneity, thanks to advanced normalization techniques like the Prescan Normalize features available on clinical scanners.

## 4 Discussion

In this study, we quantitatively compared two types of multi‐unit wireless resonators—strongly coupled and highly decoupled. Our results demonstrate that decoupled wireless resonators consistently outperform coupled ones in both SNR and g‐factor metrics. Notably, the decoupled resonator even improved parallel imaging performance relative to the original receive array, suggesting that it may offer a practical and effective enhancement for existing systems.

While it is true that high inter‐channel noise correlation is typically associated with an increased g‐factor, this is only the case if the coils' sensitivity maps are similar. In the presence of wireless resonators, the receive sensitivity maps of some coils in the head array are dominated by the wireless resonators, thereby becoming significantly different from the original sensitivity maps. This difference helps preserve distinct and complementary sensitivity profiles across the array, which can enhance spatial encoding and improve parallel imaging performance. Therefore, our findings demonstrate that the decoupled‐unit wireless resonator design reduces the g‐factor compared to the original array, even though the noise correlation values are slightly higher.

The 3‐loop resonator array presented in this work is highly decoupled, as each pair of elements is isolated either through geometric overlap or by a capacitive decoupling network. The high level of isolation among these resonators is demonstrated in Figure S2. Maintaining high isolation between units becomes increasingly challenging as the number of wireless resonator units grows. Unlike conventional coil arrays, wireless resonators cannot rely on preamplifier decoupling, making traditional methods ineffective. As such, more advanced solutions, such self‐decoupling technique [44], are needed to ensure high performance.

In addition to receiving performance, detuning is critical for the integration of wireless resonators into clinical MRI systems. Since the body coil provides a well‐calibrated and homogeneous B_1_^+^ field, wireless resonators are expected to enhance reception without interfering with transmission. Otherwise, treating them as local transmit coils would require extensive modifications and changes to scanner‐specific coil settings, which are typically impractical in clinical environments.

Figure 2 confirms that both resonator types are effectively transparent during transmission and do not affect the body coil's B_1_^+^ performance under default scanner settings—an important consideration for clinical use. However, decoupled structures offer a simpler implementation, as each unit can be independently detuned to remain transparent to the RF field. In contrast, coupled resonators present multiple resonant modes, all of which must be suppressed during the transmit phase. It should also be noted that passive detuning may not be enabled if the transmit power is extremely low, such as at a 1° flip angle. Based on our experience, a flip angle greater than 5° is generally sufficient to activate passive detuning, and most MRI pulse sequences employ flip angles well above the threshold.

At 1.5 T, head/neck coils typically use 16–20 large elements, leaving room for local SNR gains and reducing the risk of over‐coupling with the wireless resonator. Bench tests suggest that the resonance peak of 3‐loop resonator array near the Larmor frequency is still partially preserved inside the unplugged head array (Figure S3). At higher fields (3 and 7 T), head coils usually employ 32 smaller elements, and future studies are needed to determine whether similar improvements can be achieved under these conditions. An increase in local SNR generally translates into higher temporal SNR (tSNR) in fMRI, improving sensitivity to BOLD changes [45, 46]. Accordingly, beyond structural imaging, the proposed wireless resonators may enhance fMRI by boosting cortical SNR. Consistent with this, Figure S4 shows measured tSNR maps from 50 repeated GRE images on the phantom that mirror the SNR improvements. Although phantom GRE does not capture human cortical fMRI confounds (e.g., physiological noise), these results support the potential fMRI benefit of wireless resonators and motivate future in vivo validation.

This study has several limitations. First, the observed benefits are limited to local SNR improvements, with no significant changes in central SNR. Second, the evaluation was restricted to partial‐volume wireless resonators; volume‐type arrays warrant further investigation. Third, the tested resonator units were much smaller than the coil elements, minimizing frequency shifts when integrated. However, if frequency shifts occur during tuning, we recommend adjusting slightly above (< 5%) rather than below the Larmor frequency. Fourth, direct electric (E−) field or temperature measurements were not performed. However, our previous simulations [26, 27] demonstrated that there is no E‐field distortion when the wireless resonators are properly detuned. Fifth, in this study, the distance between the wireless resonators and the head coil was not optimized; they were placed ∼1 cm away, determined by the thickness of the available MRI‐compatible pad. However, if the elements of the primary coil and the wireless resonator unit are of similar size, larger spacing may be required to avoid over‐coupling.

## 5 Conclusion

This study demonstrates that decoupled wireless resonators significantly enhance SNR and parallel imaging performance compared to coupled designs when used as passive inserts in MRI receive arrays. Both decoupled‐unit and coupled‐unit designs provide sufficient detuning performance, ensuring transparency to the RF field during the transmit phase and making them viable for practical clinical MRI applications. Decoupled wireless resonators offer a cost‐effective way to improve the performance of existing receive arrays without increasing the number of array elements, which can be expensive and bulky. Future research should focus on advancing decoupling techniques and exploring their applications in more complex, multi‐dimensional wireless coil arrays to further enhance imaging performance and clinical utility.

## Conflicts of Interest

Haoqin Zhu is currently employed by Sino Canada Health Institute. The other authors declare no conflicts of interest.

## Supporting information


**Figure S1:** Measured SNR of the 3‐unit coupled resonator, as well as the 7‐unit coupled and 3‐unit decoupled resonators: (A) Axial SNR maps of the 12‐channel head array alone, with the wireless 3‐unit ladder resonator, with the wireless 7‐unit ladder resonator, and with the wireless 3‐loop resonator. (B) Photograph of the fabricated 3‐unit ladder resonator. (C) One‐dimensional (1D) SNR profiles along the indicated direction (white dotted lines in A).
**Figure S2:** Double‐probe (S21) measurements of a single wireless resonator (without the presence of other resonators) and a 3‐loop decoupled wireless resonator array. No peak splitting is observed, and the high unloaded Q‐factor demonstrates excellent decoupling. The unloaded Q‐factor remains comparable to that of a single ideal loop (238 vs. 260).
**Figure S3:** Measured S21 plots of a double pick‐up probe for the 3‐loop wireless resonator, obtained outside (left) or inside (right) the commercial head array (unplugged; preamplifiers inactive). When placed inside the array, the resonance response changed, yet a primary peak remained near the Larmor frequency.
**Figure S4:** Measured axial tSNR maps from single‐slice GRE scans. In this setup, the wireless resonator, when applied, was positioned on top of the cylindrical phantom. GRE parameters: FOV = 200 × 200 mm^2^; slice thickness = 5 mm; TR/TE = 100/10 ms; FA = 25°; matrix = 128 × 128; bandwidth = 260 Hz/pixel; 50 repeats; acquisition time per scan = ∼12 s. Voxel‐wise tSNR was computed as the temporal mean divided by the temporal standard deviation across the time series. The tSNR maps mirror the SNR results: (1) both the wireless ladder resonator and the 3‐loop resonator improved tSNR relative to the head array alone; and (2) the 3‐loop resonator provided a larger gain. Because this is a phantom experiment, the results may not fully reflect cortical fMRI in humans (e.g., physiological noise). Nevertheless, the data indicates that SNR gains translate into higher tSNR, suggesting potential benefits for cortical fMRI.

## Data Availability

The data that support the findings of this study are available from the corresponding author upon reasonable request.
