# Supplementary material for: Wireless Resonators With Coupled Versus Decoupled Units: Which Enhances Local SNR of RF Receive Arrays Better?
Source: Magn Reson Med. 2025 Nov 5;95(4):2442–50. doi: 10.1002/mrm.70170 (PMC12850623; doi:10.1002/mrm.70170)
Supplement: Supplementary file 1 — Figure S1: Measured SNR of the 3‐unit coupled resonator, as well as the 7‐unit coupled and 3‐unit decoupled resonators: (A) Axial SNR maps of the 12‐channel head array alone, with the wireless 3‐unit ladder resonator, with the wireless 7‐unit ladder resonator, and with the wireless 3‐loop resonator. (B) Photograph of the fabricated 3‐unit ladder resonator. (C) One‐dimensional (1D) SNR profiles along the indicated direction (white dotted lines in A). Figure S2: Double‐probe (S21) measurements of a single wireless resonator (without the presence of other resonators) and a 3‐loop decoupled wireless resonator array. No peak splitting is observed, and the high unloaded Q‐factor demonstrates excellent decoupling. The unloaded Q‐factor remains comparable to that of a single ideal loop (238 vs. 260). Figure S3: Measured S21 plots of a double pick‐up probe for the 3‐loop wireless resonator, obtained outside (left) or inside (right) the commercial head array (unplugged; preamplifiers inactive). When placed inside the array, the resonance response changed, yet a primary peak remained near the Larmor frequency. Figure S4: Measured axial tSNR maps from single‐slice GRE scans. In this setup, the wireless resonator, when applied, was positioned on top of the cylindrical phantom. GRE parameters: FOV = 200 × 200 mm2; slice thickness = 5 mm; TR/TE = 100/10 ms; FA = 25°; matrix = 128 × 128; bandwidth = 260 Hz/pixel; 50 repeats; acquisition time per scan = ∼12 s. Voxel‐wise tSNR was computed as the temporal mean divided by the temporal standard deviation across the time series. The tSNR maps mirror the SNR results: (1) both the wireless ladder resonator and the 3‐loop resonator improved tSNR relative to the head array alone; and (2) the 3‐loop resonator provided a larger gain. Because this is a phantom experiment, the results may not fully reflect cortical fMRI in humans (e.g., physiological noise). Nevertheless, the data indicates that SNR gains translate into higher tSNR, suggesting [file MRM-95-2442-s001.pdf]

Supplementary Materials

The Supplementary Information includes 4 Supplementary Figures.

Wireless Resonators with Coupled vs. Decoupled Units: Which Enhances Local SNR of RF Receive Arrays Better?

Ming Lu [a,b], *Haoqin Zhu* [c], *Ruilin Wang* [b], *Xinqiang Yan* [a,d,e]

a. Vanderbilt University Institute of Imaging Science, Vanderbilt University Medical Center, Nashville, TN, 37232, USA
b. College of Nuclear Equipment and Nuclear Engineering, Yantai University, Yantai, Shandong, China
c. Sino Canada Health Institute Inc., Winnipeg, Manitoba, Canada
d. Department of Radiology and Radiological Sciences, Vanderbilt University Medical Center, Nashville, TN, 37232, USA
e. Department of Electrical and Computer Engineering, Vanderbilt University, Nashville, TN, 37232, USA

Corresponding Author:

Xinqiang Yan, Ph.D.
Vanderbilt University Institute of Imaging Science
1161 21st Avenue South
Medical Center North, D-2205
Nashville, TN 37232-2310
Phone No: 1(615) 5253989
Email: xinqiang.yan@vumc.org

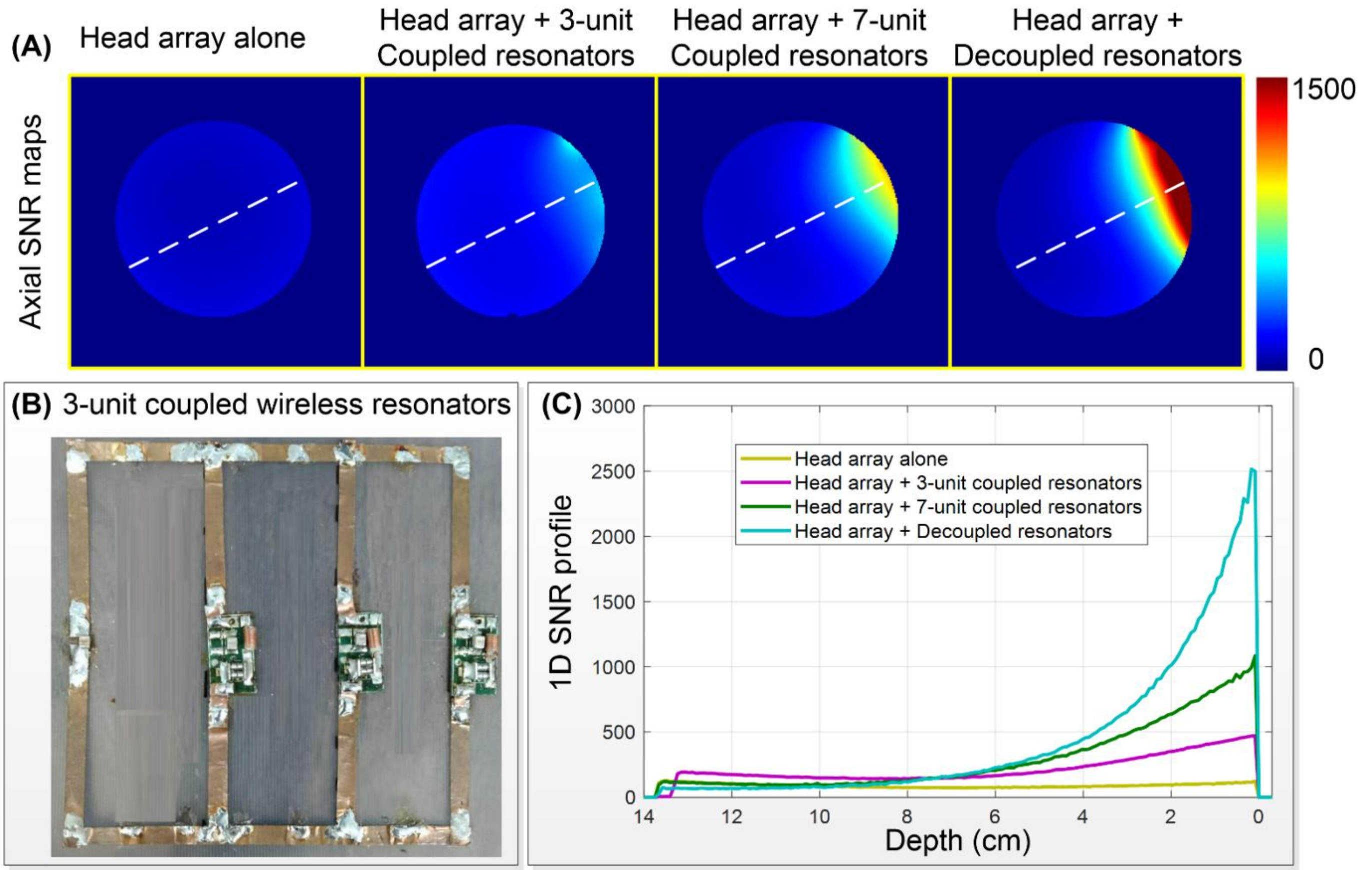

Figure S1 Measured SNR of the 3-unit coupled resonator, as well as the 7-unit coupled and 3-unit decoupled resonators: (A) Axial SNR maps of the 12-channel head array alone, with the wireless 3-unit ladder resonator, with the wireless 7-unit ladder resonator, and with the wireless 3-loop resonator. (B) Photograph of the fabricated 3-unit ladder resonator. (C) One-dimensional (1D) SNR profiles along the indicated direction (white dotted lines in Figure S1A).

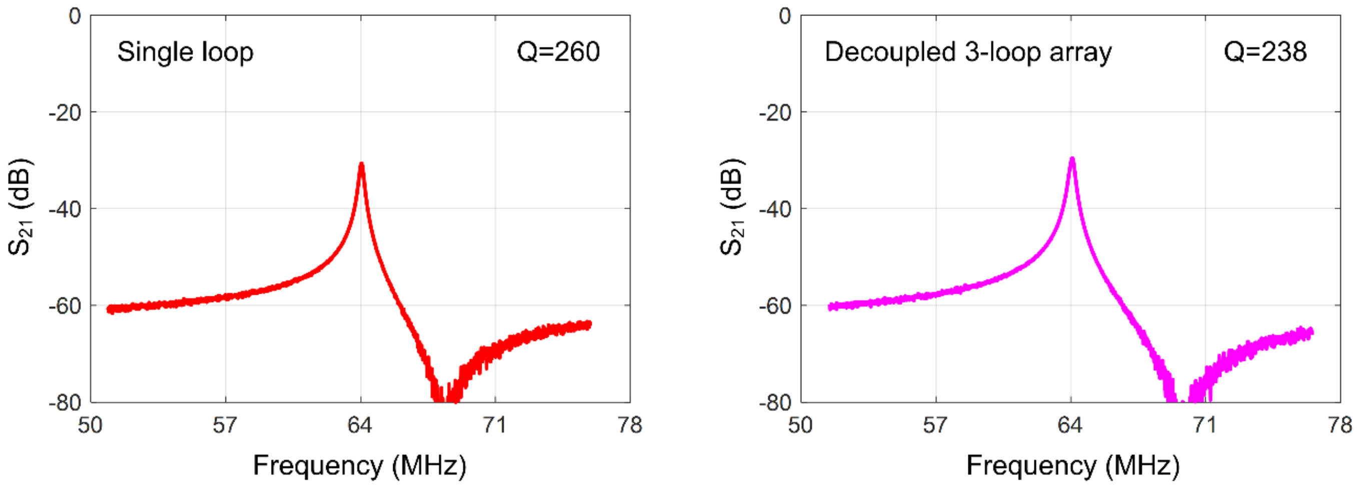

Figure S2 Double-probe (S_{21}) measurements of a single wireless resonator (without the presence of other resonators) and a 3-loop decoupled wireless resonator array. No peak splitting is observed, and the high unloaded Q-factor demonstrates excellent decoupling. The unloaded Q-factor remains comparable to that of a single ideal loop (238 vs. 260).

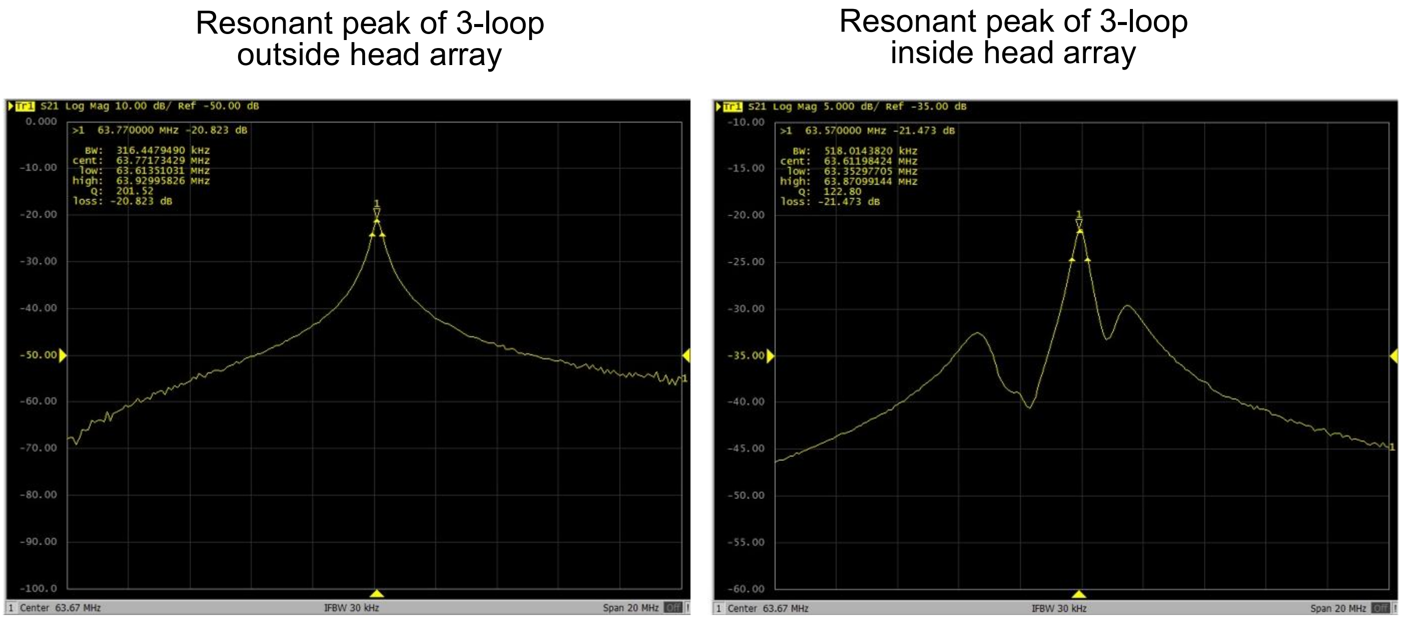

Figure S3 Measured S_{21} plots of a double pick-up probe for the 3-loop wireless resonator, obtained outside (left) or inside (right) the commercial head array (unplugged; preamplifiers inactive). When placed inside the array, the resonance response changed, yet a primary peak remained near the Larmor frequency.

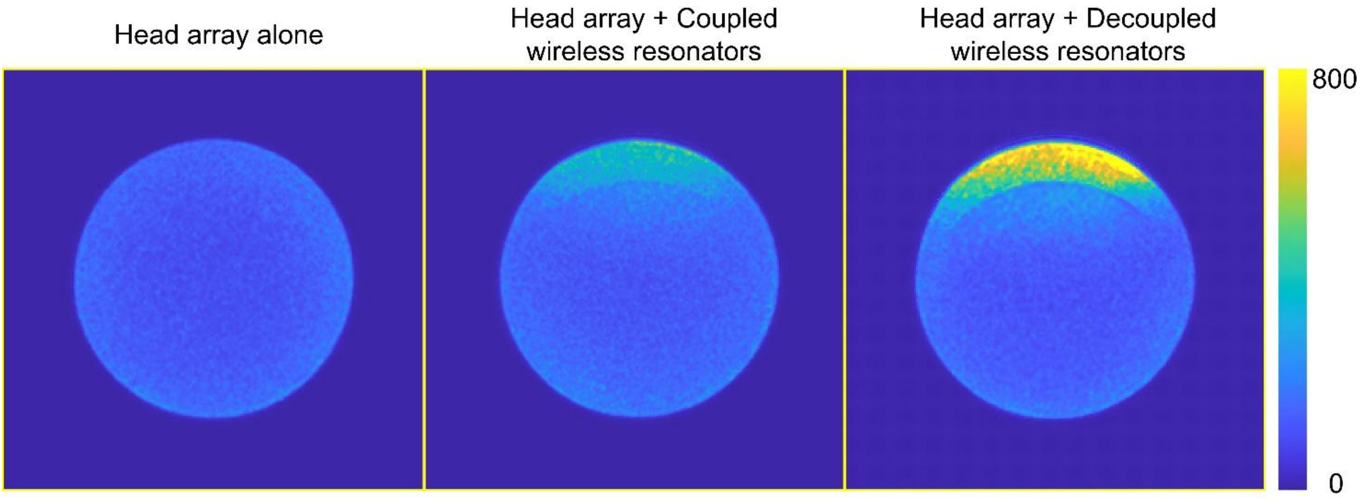

Figure S4 Measured axial tSNR maps from single-slice GRE scans. In this setup, the wireless resonator, when applied, was positioned on top of the cylindrical phantom. GRE parameters: FOV = 200 × 200 mm^2; slice thickness = 5 mm; TR/TE = 100/10 ms; FA = 25°; matrix = 128 × 128; bandwidth = 260 Hz/pixel; 50 repeats; acquisition time per scan = ~12 s. Voxel-wise tSNR was computed as the temporal mean divided by the temporal standard deviation across the time series. The tSNR maps mirror the SNR results: (1) both the wireless ladder resonator and the 3-loop resonator improved tSNR relative to the head array alone; and (2) the 3-loop resonator provided a larger gain. Because this is a phantom experiment, the results may not fully reflect cortical fMRI in humans (e.g., physiological noise). Nevertheless, the data indicates that SNR gains translate into higher tSNR, suggesting potential benefits for cortical fMRI.
